# The Glasgow Cancer Hospital (Free)

**Published:** 1909-09-18

**Authors:** 


### HOSPITAL ADMINISTRATION.

### CONSTRUCTION AND ECONOMICS.

### THE GLASGOW CANCER HOSPITAL (FREE).

DESCRIPTION OF NEW BUILDINGS.

For a very long time the supporters of the Glasgow Cancer Hospital have realised that in order to carry out the work the hospital would require to be rebuilt, or more extensive premises acquired in another part of the city. The site of the old buildings in Garnethill on one of the highest eminences of the city was considered an ideal one, and after the managers had consulted with the staff they decided that the solution of the difficulty was to rebuild the hospital on the present site.

The portion now to be opened for patients comprises four large wards, each containing eight beds. These wards are lighted from three sides, the principal light being from the south. Although the site is a limited one, the buildings have been so arranged that the wards to the north project beyond the south wards, have the advantage of receiving a south light, and have also a south balcony.

In addition to the wards for eight beds, nine single rooms have been provided, all with a southern exposure. These will be used for patients requiring isolation. A convalescent ward has also been provided. All sanitary annexes are contained in towers, which are entirely cut off from the wards, and are fitted up with the most approved pattern of baths, lavatories, and basins. Various fittings and furnishings specially suited for the particular class of patients to be treated have been installed, which should add very materially to the convenience in nursing and comfort of the patients. On each flat a large surgical dressing-room is provided, fitted up with suitable basins, sterilisers, etc., so that all surgical dressings may be carried out in this room.

The beds are of the "Balcony" pattern, so that patients can be removed from the ward to the surgical dressing-room and wheeled back again to the ward with the least possible inconvenience and the minimum of discomfort. An operating theatre will be provided on each ward level.

The top of the building has been fitted up as a research department. The light here is from the north, and as the building stands on a very high eminence, the light is ideal for a city site. On the basement is placed a fully equipped kitchen, which is ventilated by means of an electric fan, so that the odour of cooking will not permeate any part of the hospital.

The nurses' home occupies the western portion of the site. Each nurse is provided with a separate bedroom. There are also dining-rooms, sitting-rooms, etc., along with the suite of rooms for the matron.

Behind the main building a complete and up-to-date laundry has been erected, in which the most modern machinery has been installed, worked by electricity, and, in addition, there is provided a destructor and steam disinfector. Quite detached from the main building a small mortuary and mortuary chapel have been erected.

The buildings are lighted by electricity, and are heated on the "Reck" system by means of radiators. Each radiator is under direct control, and has a supply of cold air guarded by a regulator, so that any portion of the building may be heated to the required temperature. There is a complete installation of telephones.

The patients will shortly be removed from the old portion of the building into the new wards, in order that the older portions remaining may be entirely rebuilt, and in six months it is hoped the whole building will be finished. The entire buildings will then be on modern lines, as they are designed in every detail for the particular class of work which is to be carried out. The directors confidently believe that when the Glasgow public fully appreciate the boon which such an institution will be to the community of Glasgow, the needed support for its upkeep will be forthcoming.

The entire buildings and equipment have been carried out under the supervision of Dr. D. J. Mackintosh, superintendent of the Western Infirmary, Glasgow, and by Messrs. James M. Monro and Son, architects, Glasgow. Sir George T. Beatson, K.C.B., M.D., who is the senior surgeon of the staff, has taken a deep interest in the whole scheme, having acted as chairman of the Building Committee.

